# Negative effects of the SARS-CoV-2 pandemic: The interlinking of maternal attachment representation, coping strategies, parental behavior, and the child's mental health

**DOI:** 10.3389/fped.2022.939538

**Published:** 2022-11-01

**Authors:** Manuela Gulde, Franziska Köhler-Dauner, Inka Mayer, Ute Ziegenhain, Jörg M. Fegert, Anna Buchheim

**Affiliations:** ^1^ Department of Child and Adolescent Psychiatry/Psychotherapy, University of Ulm, Ulm, Germany; ^2^ Institute of Psychology, University of Innsbruck, Innsbruck, Austria

**Keywords:** SARS-CoV-2 pandemic, attachment representation, coping, child's mental health, childhood maltreatment, parental behavior

## Abstract

For more than two years, young families have been confronted with a large number of restrictions and following burdens as a result of the SARS-CoV-2 pandemic. In fact, it became evident, that the current circumstances are particularly stressful for child's mental health. With regard to the child's mental health in times of a pandemic, additional factors within the family, such as maternal attachment representations as well as coping strategies and parental behavior, may play an important role. This study aims to investigate the interplay of maternal attachment representation, coping strategies, parental behavior and child's mental health during the SARS-CoV-2 pandemic. In this longitudinal study, previously collected data regarding maternal attachment representation and newly attained data from the SARS-CoV-2-pandemic-assesment (lack of coping strategies, children's mental health and parental behavior) were combined and analyzed. The data were collected in an online survey since beginning of the pandemic, including *N *= 73 mothers. A path model was calculated in form of multiple linear regression. A path model could be confirmed, which indicates that insecure maternal attachment representation predicts lack of coping strategies during the pandemic [*b* = 5.55, 95%-CI = (4.51; 6.55), *p* = 0.001]. Furthermore, lack of coping strategies predicts harmful parental behavior during the pandemic [*b* = −0.77, 95%-CI = (−1.27; −0.21), *p* = 0.007], which in turn predicts children's mental health problems, namely behavioral problems [*b* = −0.08, 95%-CI = (−0.14; −0.01), *p* = 0.027]. Presence of short-time work and decrease in income since beginning of the pandemic were used as control variables. This means that since the pandemic mothers with insecure attachment representation have an increased risk of having only a few coping mechanisms available, leading to harmful parental behaviors and ultimately affecting the mental health of their children. In conclusion, the pandemic could potentially have a particularly negative influence on mothers with an insecure attachment type and therefore on their children. Therefore, tailored interventions for families should be offered that both focus on the different types of mental health problems in children and support parents in their coping skills.

## Introduction

For over two years the world's population has been facing the numerous restrictions and challenges caused by the current severe acute respiratory syndrome coronavirus type 2 (SARS-CoV-2) pandemic. These restraints include the endorsement of social distancing, sudden closure of schools and childcare, the loss of community programs and jobs, an increase in recessions or unemployment, home schooling, as well as lack of social support, for example from grandparents (1–4). Particularly families with young children are seriously affected by these measures and problems and in need to find solutions. Even important face-to-face meetings among children and young people in organized leisure activities like sports clubs, church or band practices are only very selectively possible due to social distancing and closures. This means crucial and development-relevant parameters of everyday life suddenly break away with potentially social, emotional and cognitive effects (5–7).

These consequences may be more urgently felt among children that are lacking a stable parental home. Numerous studies have previously shown that particularly in times of stress and uncertainty, that characterize a crisis such as the current SARS-CoV-2 pandemic, especially young children urgently need a secure and stable family environment (8–11). We may assume that a stable parental home seems to be a relevant protective factor in times of a pandemic. Since the parental role may hold a challenge for some mothers and fathers even in non-pandemic times it becomes evident that not all parents may be able to meet the special needs of their children in the demanding times of a pandemic (12). Parents confronted with psychological stress may have limited resources to recognize their children's needs (13).

The way individuals deal with stress or stressful live-events is closely linked to their attachment representation. Previous research shows that different attachment representations are related to various coping strategies, which can increase, prolong or improve stress responses (14–16).

The attachment system is the individual's homeostatic mechanism for regulating distress. It is developed in early childhood but is hypothesized to continue to influence emotional regulation and functioning throughout the entire lifespan. For example, if caregivers are insensitive, unresponsive or inconsistently available, the individual develops alternative methods to regulate affect. This can manifest from hypervigilance to signs of rejection or separation and a tendency to be overwhelmed by negative affect or to exaggerate distress in order to elicit a helping response in others. Therefore, adults with insecure attachment representations could cause ill-organized working models that are distorted and disrupted by defensive processes that frequently stand in the way of successful, engaging coping (17). Secure attachment style functions as a protective factor while coping with stress and depressive symptoms and people with secure attachment style are more likely to effectively regulate their negative emotions and have better strategies for solving problems when they experience fear and threats (18), i.e., sought social support in stressful situations more often than adults with insecure attachment (19).

Despite divergent definitions and conceptualizations, coping can generally be understood as a response to stressful situations with the aim of psychosocial adjustment (20). There are cognitive or behavioral ways to cope with stressors (21). Cognitive coping strategies aim to change one's perception or appraisal of a situation, whereas behavioral coping skills refer to actions which reduce the resulting effects of stressors, such as arising distress. According to Carver and Connor-Smith (22) these cognitive or behavioral strategies can further be divided into engagement and disengagement coping. Dijkstra and Homan (23) stated that engaging coping strategies such as confronting rather than diverting from stressors or their effects led to higher perceived control. In contrary, disengaging strategies induced a lack of control and were found to be related to deteriorated psychological well-being (23).

There is also evidence for an association between adult attachment, coping and parental behavior. Branjerdporn and colleagues (24) found that insecure attachment correlates with high levels of sensory sensitivity, which is associated with the use of passive coping strategies rather than active coping strategies when facing stressors. They also found a significant relation between adult anxious attachment and authoritarian as well as permissive parental behavior which was partially mediated by sensory sensitivity. Besides, adult attachment avoidance was related to permissive parental behavior. This relation was fully mediated by sensory sensitivity.

The pandemic is a stressful time for all families. Findings show that families with risk factors, such as parents with insecure attachment representation, are particularly at risk of not coping as well during the pandemic as families with low risk factors (25–27). Specifically, adaptive coping strategies, secure attachment representation and supportive family environments may serve as protective factors for families experiencing stress and may differentially influence abuse potential (23).

Therefore, the aim of our study was to analyze the pathways between such risk factors, namely the maternal quality of attachment representation, the ability of coping, parental behavior and hence the mental health of the child during the SARS-CoV-2-pandemic.

## Methods

### Study design

TransGen is a joint interdisciplinary project with the goal to investigate protective and risk factors regarding the transgenerational transmission of maternal maltreatment experiences. In a prospective study design data comprising psychological, biological, and social factors from mothers and their new born child were collected. Five subprojects, including four clinical studies and one animal model, are part of the TransGen joint interdisciplinary project. From October 2013 until March 2017 data for this study has been collected. The project was funded by the Federal Ministry of Education and Research and it was approved by the University Ethics Committee Ulm.

Mother-child-dyads were recruited at the maternity unit of the Ulm University Hospital and accompanied during the first years of the child's life. Within three days after birth (measurement time *t*_0_) the German version of the Childhood Trauma Questionnaire (CTQ) (28, 29) was used to access the maternal childhood maltreatment experiences. We also collected more data in three follow-up measurements: three months after birth (*t*_1_), twelve months after birth (*t*_2_), and roughly three years after birth (*t*_3_).

To measure the current stress level of the mothers due to the pandemic, mothers participated in two online “SARS-CoV-2 pandemic surveys” in two periods of time. The first lasting from May 18th until July 31st, 2020, the second from March 1st until May 31st, 2021. The following results refer to the data from the second time slot only. For the online survey the platform “Unipark” was used. For all mothers in the study from 2013 to 2017 a profile was created with pseudonym in order to be able to assign the answers to the respective mothers in the course of the study. All participants received the same link to the survey by e-mail and only had to enter their individual pseudonym to connect their survey answers with their previous study data. The online “SARS-CoV-2 pandemic surveys was constructed in such a way that the survey could only be completed if all questions were answered and all necessary information was provided.

### Participants

Between October 2013 and December 2015, a total of 533 mother-child-dyads were recruited in the maternity unit of the University Hospital of Ulm shortly after birth. Participants were excluded if any of the following exclusion criteria were fulfilled: Insufficient knowledge of the German language, mother's age <18, mother's current or former drug or alcohol abuse, mother's poor health (e.g., AIDS disease, hepatitis, etc.) or severe mental illness, child's extremely low birth weight (less than 1500 g), serious premature birth (less than 37 weeks of pregnancy) or birth complications. Written informed consent was provided by 240 mothers. The dyads were invited to a first laboratory and home visit data assessment 3 months postpartum (t1: laboratory and home visit), where maternal attachment representation was assessed. A total of *N* = 240 mother-child-dyads participated in the study at this assessment point. A second laboratory and home visit around the age of 12 months (*t*_2_) were attended by 158 mother-child-dyads. These pairings also attended the third data collection around the child's third birthday (*t*_3_). The 158 dyads were then contacted again per mail and asked to participate in the additional online questionnaire “SARS-CoV-2 pandemic survey” concerning the effect of the pandemic on families. 91 of the contacted mothers were willing to edit the survey until the end of July 2020. There were different reasons for not participating in measurement like a lack of time, no willingness to take part in a particular survey concerning the SARS-CoV-2 pandemic or merely not reaching the families. The second online “SARS-CoV-2 pandemic” survey was filled out by *n *= 73 participating mothers by the 31st of May 2021, where consequences of the SARS-CoV-2, coping strategies and parental behavior were assessed. We concluded a total of two waves of data collection (*t*_0_*: May 18th–July 31st, 2020, *t*_1_*: March 1st–May 31st, 2021), where the same measurements were collected. Analysis was just executed for complete data sets of mother-child-dyads at every wave of data collection, resulting in *N* = 73 sets after excluding missing values.

### Measures

#### Consequences of SARS-CoV-2

In the “SARS-CoV-2-pandemic survey”, which was collected in an online survey, numerous socio-demographic data of the mothers and their families were assessed. These included age, educational level, occupations, and marital status, as well as the number of minors living in the household and the number of own children. Furthermore, more information regarding the mothers’ and her potential partners' employment was examined. We also asked whether they were currently working in an essential field of work, whether they experienced short time work since the beginning of the pandemic, and whether the household's income had decreased by more than a quarter.

#### Maternal attachment

Maternal attachment representations were assessed at t1 using the Adult Attachment Projective Picture System (AAP) (30). The AAP is a standardized, objective, reliable and valid attachment interview using eight line-drawings. The participants are shown these drawings and asked a standardized set of questions to tell a story to each picture. The first is a neutral warm-up picture, which is followed by seven drawings depicting attachment-related scenes (e.g., separation, illness, loss, potential maltreatment). These seven stimuli are designed to activate the participant's attachment system. The participant's audio-recorded responses are evaluated considering content, discourse and defensive processes along the manual (30). In the AAP, the attachment representation is expressed by assigning it to one of the four attachment classifications: “Secure attachment”, “Insecure-distant attachment”, “Insecure-entangled attachment” and “Unprocessed trauma” (30). However, since in our study the N in the individual attachment classifications was too low to evaluate them individually, this study only distinguished between “secure attachment” and all other classifications which were summarized under the term “insecure attachment”. Therefore, only the two superordinate classes are referred to in the analysis of the data of this study. All interviews were conducted by trained psychologists. AAP classifications were coded by two independent certified judges. Inter-rater reliability showed significant concordance for the four-group classification (*κ *= 0.95, 95%-confidence interval [0.88, 1.04], *p *< 0.001), and for the two-group classification (organized vs. unresolved, *κ *= 0.96), 95%-confidence interval [0.91, 1.00], *p *< 0.001.These data are also consistent with results of validation studies on AAP. In the extensive psychometric validation study by George & West (30), the agreement between the AAP and AAI categories, the interrater reliability, the test-retest reliability (interval: three months) and the discriminant validity were checked. The reliability and validity of the AAP could be confirmed in the results of this study with an agreement of 90% between AAP and AAI regarding the four most relevant attachment groups. Interrater reliability was tested in this validation study between one primary rater and two independent raters. In this study, agreement between rater 1 and 2 was 90% (*κ *= 0.79, *p* = 0.000) for the 4-group classification, and 85% (*κ *= 0.79, *p* = 0.000) between rater 1 and 3. For the 2-group classification, the agreement was 99% (*κ *= 0.66, *p* = 0.000) and 85% (*κ *= 0.79, *p* = 0.000). These results indicate a concurrent validity of the results of the study with the AAI.

The AAP classifies the four established attachment categories: secure, insecure-dismissing, insecure-preoccupied, and unresolved attachment. For our present study, attachment representations of the mothers were divided into two major classifications “secure” (F) and “insecure” (insecure-dismissing (Ds), insecure-preoccupied (E) and the unresolved attachment status (U).

#### Coping strategies

Psychological coping resources were measured in an online survey during the pandemic using the Pearlin Mastery Scale (31), considering individual resources and flexibility or perceived control. The scale consists of 7 items on a 4-point-Likert scale (1 = “strongly disagree” to 4 = “strongly agree”) and therefore has a range from 7 to 28 points. The higher the score, the greater is the inability to exert coping strategies. A higher score means a greater tendency to have a lack of individual coping strategies. Hence our operationalization of coping strategies refers to the degree of perceived control over one's life, which reflects the individual's ability to effectively handle stressful situations or to execute appropriate strategies in dealing whit these situations. For example, a low score on the Pearlin Mastery Scale would mean that a person feels they have no control over important things in their life.

#### Parental behavior

We used 4 items in an online survey during the pandemic to measure whether there is an increase in harmful parental behavior since beginning of the pandemic. The items are in detail: “I've been yelling at the child more”, “I am more impatient with the child”, “Everyday life with the child is very chaotic”, “I experience increased fear of raising my hand against the child”. The items were rated on a 7-point Likert scale (1 = “does not apply at all” to 7 = “applies very much”) with a higher score indicating a more pronounced harmful parental behavior. This means, the total item score states the extent of change in harmful parental behavior during the pandemic. Cronbachs Alpha was measured at *α* = 0.84.

#### Children's mental health

The Children's mental health was assessed in an online survey using the German version of the Strengths and Difficulties Questionnaire (SDQ) (32), a behavioral screening questionnaire which is filled in by a parent. This instrument consists of five scales (emotional problems, externalizing behavioral problems, hyperactivity/attention problems, problems with peers and prosocial behavior) addressing positive and negative behavioral attributes of the children. Each scale contains 5 items and is rated on a 3-point Likert scale. In the “SARS-CoV-2-pandemic survey” a selection of these items was included. For the emotional problems scale all five items were included: “Often complains of headaches, stomach-aches or sickness”, “Many worries or often seems worried”, “Often unhappy, depressed or tearful”, “Nervous or clingy in new situations, easily loses confidence”; and “Many fears, easily scared”. For the externalizing behavioral problems scale the following two items were chosen: “Often loses temper” and “Generally well behaved, usually does what adults request”. The questions “Restless, overactive, cannot stay still for long” and “Constantly fidgeting or squirming” were selected as items for the hyperactivity/attention problems scale. In order to operationalize, for each of these three scales the individual item-values were summed up. The two scales “problems with peers” and “prosocial behavior” were not included, because of the children's limited social contacts outside of the family due to pandemic-related restrictions regarding school and kindergarten.

## Statistical analyses

For all analyses significance level was defined with.05 as the critical alpha level. The data were evaluated using the SPSS Statistics 24.0 program (33). Mothers could not be supervised when answering the items of the online survey, therefore some questionnaires weren't completed. Only complete data sets were used for the data analysis. Descriptive statistics with means, standard deviations and relative frequencies are reported. Before considering the hypotheses descriptive statistics and two tailed Pearson correlations of model and control variables were calculated. Model variables were attachment representation using the AAP, average lack of coping strategies, average of harmful parental behavior, as well as the SDQ sum scores of the subscales hyperactivity, externalizing problems, and emotional problems. Presence of short-time work (coded as 1 = short-time-work, 2 = no short-time-work) and decrease in income (1 = decrease in income, 2 = no decrease in income) were included as control variables and were measured at both online-surveys.

Subsequently, the paths of the assumed path model were calculated using multiple linear regressions. The order of the variables was, as already mentioned, as following: maternal attachment representation, average lack of coping strategies, average of harmful parental behavior, and SDQ sum scores of the subscales hyperactivity, externalizing problems, and emotional problems. A total of three regression models were calculated, where the third model was calculated with three different dependent variables (1: dependent variable: lack of coping strategies, independent variable: maternal secure attachment representation; 2: dependent variable: average of harmful parental behavior, independent variables: maternal secure attachment representation, lack of coping strategies, 3.1: dependent variable: hyperactivity, independent variables: maternal secure attachment representation, lack of coping strategies, average of harmful parental behavior; 3.2: dependent variable: externalizing problems, independent variables: maternal secure attachment representation, lack of coping strategies, average of harmful parental behavior; 3.3: dependent variable: emotional problems, independent variables: maternal secure attachment representation, lack of coping strategies, average of harmful parental behavior). Additionally, presence of short-time work and decrease in income were included as control variables.

The requirements (34) were tested using scatter plots, standardized residuals, and leverages (to check for linearity as well as for outliers), the Durbin-Watson statistic (to check for autocorrelation), the tolerance and VIF values (to check for multicollinearity), and the *P*-*P* plot (to check for normal distribution of the residuals). As no clear outliers were determined based on more than one of the several criteria used, no individuals were excluded from the data analysis. Since heteroscedasticity was seen on visual inspection of the scatter plots, the regression analysis was performed with 1,000-fold bootstrapping to avoid bias in the coefficients. All other prerequisites were met.

## Results

### Descriptive analyses

Descriptive analyses are presented in Table 1. *N* = 73 mothers completed the second online survey of the SARS-CoV-2 online survey. The average age of the mothers was *M *= 38.4 years old (*SD *= 4.0), with a range of 31 to 46 years. 64.4% of the women had a high school diploma, 13.7% had a secondary school diploma, and 19.2% had a lower secondary school diploma. Only 2.7% asserted that they did not have a high school diploma. 26% of the mothers at the first measurement point and 11% at the second measurement point stated to be affected of short-time work. At the first survey 46.6% and at the second 5.5% reported a decrease in income since beginning of the pandemic. The children were between 4 and 8 years old on average *M *= 5.3 years (*SD *= 1.1). There was an equal gender distribution among the children.

**Table 1:** Descriptive analysis.

	*M*	SD	Range
Mother's age	38.4	4.0	31–46
Children's age	5.3	1.1	4–8
* *	*N*	%	
Education			
High school diploma	47	64.4	
Secondary school diploma	10	13.7	
Lower secondary school diploma	14	19.2	
No high school diploma	2	2.7	
Affected by short-time work first measurement	19	26.0	
Affected by short-time work second measurement	8	11.0	
Decrease in income since beginning of the pandemic first measurement	34	46.6	
Decrease in income since beginning of the pandemic second measurement	4	5.5	
Insecure attachment representation	28	38.4	

Examination of the descriptive statistics revealed that 38.4% of the mothers in this sample had insecure attachment representations. The lack of coping strategies averaged at *M *= 15.5 (*SD *= 3.4) with a minimum of 8.5 and a maximum of 24.5. The harmful parental behavior ranged from 17 to 54 with an average of *M *= 36.1 (*SD *= 8.9). The sum score of the SDQ subscales were for the hyperactivity subscale at *M *= 3.5 (*SD *= 1.2; minimum = 2, maximum = 6), the externalizing problems subscale *M *= 3.6 (*SD *= 1.0; minimum = 2, maximum = 6), and the emotional problems subscale *M *= 8.3 (*SD *= 2.2; minimum = 5, maximum = 15, Cut-Off for abnormality = 5) (Table 2).

**Table 2:** Psychometric measures.

	*M*	SD	Range
Lack of coping strategies	15.5	3.4	8.5–24.5
Harmful parental behavior	36.1	8.9	17–54
Hyperactivity subscale	3.5^a^	1.2^a^	2–6^a^
Externalizing problems subscale	3.6^a^	1.0^a^	52–6^a^
Emotional problems subscale	8.3	2.2^a^	5–15

^a^ Note:only selected items of those scales were assessed, therefore comparability to other studies is limited.

### Correlation analyses

First, we demonstrate the significant correlations of the model variables: Attachment representation (coded as 0 = secure, 1 = insecure) correlated strongly and significantly with harmful parental behavior (*r *= −0.87, *p *< 0.001), lack of coping strategies (*r *= 0.79, *p *< 0.001), and emotional problems (*r *= 0.56, *p *< 0.001). Harmful parental behavior also correlated significantly with lack of coping strategies (*r *=−0.83, *p *< 0.001), hyperactivity (*r *= −0.35, *p *= 0.002), emotional problems (*r *= −0.48, *p *< 0.001) and externalizing problems (*r *= −0.31, *p = *0.008). Furthermore, lack of coping strategies correlated with hyperactivity (*r *= 0.26, *p = *0.025), externalizing problems (*r *= 0.28, *p = *0.018) and emotional problems (*r *= 0.52, *p < *0*.*001). Hyperactivity and externalizing problems also correlated significantly (*r *= 0.37, *p *< 0.001) (Table 3).

**Table 3:** Correlation analysis.

	1	2	3	4	5
1. Attachment representation					
2. Lack of coping strategies	0.79***				
3. Harmful parental behavior	−0.87***	−0.83***			
4. Hyperactivity subscale	0.22	0.26*	−0.35**		
5. Externalizing problems subscale	0.23	0.28*	−0.31**	0.37**	
6. Emotional problems subscale	0.56***	0.52***	−0.48***	0.18	0.21

***p < 0.001, **p < 0.01, *p < 0.05.

Second, correlations of the control variables are shown: Presence of short-time work at the first survey correlated with education (*r *= 0.27, *p *= 0.023) as well as short-time work at the second survey (*r *= −0.29, *p *= 0.012). Additionally, presence of short-time work at the second survey correlated significantly with lack of coping strategies (*r* = −0.26, *p *= 0.027) and decrease in income at the second measurement point (*r* = 0.30, *p* = 0.010).

### Path model

**Figure 1:**
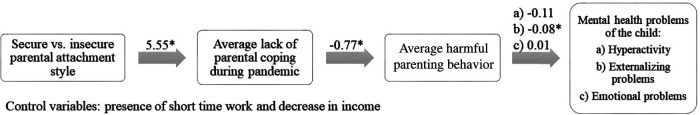
Path model of the interrelation of maternal quality of attachment representation, maternal ability of coping, harmful parental behavior and child's mental health.

The results of the multiple linear regressions used to analyze the path model are the following: (1) Lack of coping strategies were significantly predicted by secure vs. insecure attachment representations as well as presence of short-time work at the second measurement time point. (2) The harmful parental behavior was significantly determined by lack of coping strategies as well as insecure attachment representation. (3) In the final step, the child's symptoms were considered. Although it was not significant for the prediction of hyperactivity, the confidence interval (CI) of the coefficient of harmful parental behavior ends at zero, so it is still considered as a crucial variable in this model. In particular, because no other predictor showed an even remotely significant effect on hyperactivity. The child's externalizing problems were also significantly predicted only by harmful parental behavior. Emotional problems of the child, in turn, were not determined by harmful paternal behavior, as expected, but by the lack of coping strategies, which was intended to serve only as a control variable in this model. All results of the regression models to calculate the path model are shown in Table 4. The results of the path model analysis are summarized in Figure 1.

**Table 4:** Results of regression models.

Model	*b* (SE)	95%-CI	*p*	*R* ^2^_adj._
**Lack of coping**				
Constant	17.61 (1.83)	[14.20; 21.51]	0.001	
Secure attachment	5.55 (0.51)	[4.51; 6.55]	0.001	
Short-time work first survey	0.49 (0.47)	[−0.40; 1.44]	0.297	
Decrease in income first survey	0.39 (0.47)	[−0.54; 1.30]	0.412	
Short-time work second survey	−2.85 (0.93)	[−4.84; −1.17]	0.002	
Decrease in income second survey	−0.78 (0.88)	[−2.34; 1.15]	0.268	0.669
**Harmful parental behavior**				
Constant	49.81 (7.24)	[33.32; 62.22]	0.001	
Secure attachment	−11.79 (1.84)	[−15.47; −7.94]	0.001	
Lack of coping	−0.77 (0.27)	[−1.27; −0.21]	0.007	
Short-time work first survey	−1.16 (0.91)	[−3.08; 0.60]	0.204	
Decrease in income first survey	−0.55 (0.91)	[−2.30; 1.25]	0.548	
Short-time work second survey	3.03 (2.13)	[−1.01; 7.68]	0.130	
Decrease in income second survey	1.34 (2.43)	[−4.92; 5.18]	0.527	0.811
**Hyperactivity**				
Constant	13.02 (4.70)	[2.94; 21.40]	0.009	
Secure attachment	−1.55 (0.96)	[−3.25; 0.57]	0.105	
Lack of coping	0.06 (0.12)	[−0.20; 0.29]	0.588	
Harmful parental behavior	−0.11 (0.06)	[−0.22; 0.01]	0.061	
Short-time work first survey	−0.54 (0.51)	[−1.51; 0.49]	0.283	
Decrease in income first survey	−0.36 (0.44)	[−1.12; 0.58]	0.440	
Short-time work second survey	0.63 (1.03)	[−1.56; 2.38]	0.473	
Decrease in income second survey	−1.03 (1.60)	[−4.25; 2.32]	0.489	0.032
**Externalizing problems**				
Constant	11.04 (2.69)	[5.82; 16.45]	0.001	
Secure attachment	−0.83 (0.64)	[−1.88; 0.57]	0.202	
Lack of coping	0.01 (0.08)	[−0.16; 0.14]	0.935	
Harmful parental behavior	−0.08 (0.03)	[−0.14; −0.01]	0.027	
Short-time work first survey	−0.29 (0.29)	[−0.87; 0.28]	0.331	
Decrease in income first survey	0.28 (0.27)	[−0.23; 0.86]	0.339	
Short-time work second survey	−0.10 (0.72)	[−1.55; 1.36]	0.863	
Decrease in income second survey	−0.06 (0.93)	[−2.42; 1.48]	0.951	0.079
**Emotional problems**				
Constant	5.88 (5.99)	[−6.63; 16.94]	0.316	
Secure attachment	1.39 (1.43)	[−1.34; 4.36]	0.333	
Lack of coping	0.49 (0.17)	[0.18; 0.82]	0.011	
Harmful parental behavior	0.00 (0.08)	[−0.15; 0.18]	0.970	
Short-time work first survey	−0.98 (0.66)	[−2.28; 0.31]	0.147	
Decrease in income first survey	−0.48 (0.62)	[−1.61; 0.78]	0.427	
Short-time work second survey	2.41 (1.56)	[−0.57; 5.47]	0.089	
Decrease in income second survey	−0.48 (1.80)	[−4.73; 3.01]	0.753	0.393

## Discussion

The aim of our study was to investigate the relationship of maternal attachment representation, coping, parental behavior and child's mental health in the exceptional situation of the pandemic.

While many studies have already shown that regulatory measures to contain the pandemic such as contact restrictions, short-time work, school closures etc. have a negative impact on children's health and families' well-being (35, 36), we were able to show for the first time in our study that maternal attachment representation and the associated coping skills and corresponding parental behavior also significantly influence children's mental health during the pandemic.

Our path analyses partly confirmed our assumed model shown in Figure 1. Specifically, we could confirm the pathway in terms of externalizing behavior problems of the child, but also with a CI with the end just above the zero in terms of hyperactivity. Interestingly, there was no significant influence of parental behavior on emotional problems of the child. However, the lack of coping strategies had a direct influence on the child's emotional problems, which means that the intermediate step of our assumed causal chain *via* parental behavior was skipped in this case.

This effect has also been demonstrated in children of parents with cancer (37). The way in which parents with cancer cope with their illness appears to have a direct influence on the mental health of their minor children. In this context, passive-avoidant coping, as also occurs in the case of insecure attachment, seems to contribute to a higher risk of internalizing symptom formation in the children.

First, however, the three previously assumed associations (i.e., from attachment to lack of coping strategies, from lack of coping strategies to parental behavior, and from parental behavior to child mental health) are examined in more detail.

As hypothesized, maternal insecure attachment representation was associated with a greater lack of coping strategies during the pandemic. This is consistent with previous studies (14, 15, 38–40). However, in these studies different questionnaires were used to assess attachment representation, so that references back to the AAP, which captures attachment representation by an interview must be made with caution. Nevertheless all studies have demonstrated that secure attachment style functions as a protective factor while coping with stress and depressive symptoms during the pandemic and that people with secure attachment style are more likely to effectively regulate their negative emotions and have better strategies for solving problems when they experience fear and threats (18), i.e., sought social support in stressful situations more often than adults with insecure attachment (19). In our study, however, we did not focus on social support as a coping strategy but took a more generalized perspective at the lack of effective coping strategies in the form of thoughts such as “I can't cope with some of my problems.” This lack could have resulted from negative experiences, such as not seeking outside help.

Next, a negative association between lack of effective coping strategies and quality of parental behavior was assumed and confirmed. Since maternal attachment was included as a control variable in the regression, an influence of coping on parental behavior can be assumed *via* the importance of attachment representation. This significant prediction is in line with other studies. For example Levy-Shiff (41) found a relationship between appraisal patterns of stress and quality of parental behavior. We may conclude that the lower quality of parental behavior found here arises in the context of a lack of coping strategies due to being overwhelmed by demands.

In the final step of analysis, the impact of the other variables on the child's mental health were considered. In particular, externalizing behavior problems as well as hyperactivity symptoms of the child were predicted by harmful parental behavior. Previous research has shown, that conduct disorders are associated with punitive parenting strategies with the strongest effect size among several mental disorders in children (42). However, no association with emotional problems was found, which is not along our assumptions. Morris and colleagues (43) showed that emotion regulation problems in children could be improved particularly through changes in parental behaviors. In our sample, children's emotional symptoms did not show any association with parental behavior, but directly with the lack of coping strategies. This was also partially found by Wood and colleagues (44), who could not show a relationship between parenting and child anxiety. To the best of our knowledge, no research has been conducted analyzing the association between maternal coping and child emotional disturbance using a community sample. We may assume that the perception of higher demands and strain on parents in stressful times like the pandemic trigger anxiety and stress also in children.

There is a correlation in the results of this study that seems counterintuitive at first glance, yet could be a very interesting issue for future research. The data of this study suggest that parent's short-time work leads to a decrease in parenting skills and not, as one might assume, to an increase in time spent with children and thus an improvement in the parent-child relationship. This seems illogical at first, since parents on short-time work should actually have more time available for their children than, for example, parents who work from home. However, there are indeed reasons, some of which can be attributed to the specific situation during the SARS-CoV-2 pandemic in which the study was conducted. For example, short-time work during a crisis like the corona pandemic seems to be a strong threat to family income and in some cases can even threaten entire livelihoods (45). However, not only the reduced salary is a worrying factor for many parents, but also the job insecurity that comes with short-time work in such a crisis situation. Even though the short-time allowance was designed to avoid layoffs, many workers were still afraid of unemployment. In addition to the very existential and occupational factors, family and health worries may also have placed a heavy burden on parents during the pandemic and can ultimately lead to chronic stress. All these factors, individually or in sum, can negatively strain the parental behavior, for example, through the mechanism of negative parental emotions (46). At the same time, the sensitivity of the parental behavior can suffer from the psychological stress of the parents, as they themselves suffer from strong fears, burdens and stress (46). It can also be assumed that parents spend less time with their children, although they would actually have more time to spend with them. However, because they are so busy with their own emotions and thoughts, they cannot use this free time as time with their children, but need it for themselves. These are some possible explanations that can be considered to explain the unexpected connection between short-time work and parent-child relationships, but further research is needed to understand this connection.

### Limitations

We have to consider several limitations in the present study: First, the sample is restricted to participants from an online survey, which inhibits the generalization and the sample cannot be considered representative (47). Moreover, due to the short time period of data collection at the beginning of the pandemic we were working with a relatively small sample size of *N* = 73 participants. Therefore, statistical power of the results might be limited (48). Further research should investigate models with a larger sample size to verify the results. In addition, our sample includes a high percentage of the mothers with a high level of education, which has not been reported in other German cohort studies (49, 50). This might limit the representativeness of the study. In addition, the use of self-report measurements for maternal coping strategies, parental behavior as well as child's psychological health may lead to biased answers due to social desirability. There are currently no studies that show correlations between the attachment status and self-reported attachment style measured by interview methods in the AAP, therefore it must be emphasized in this study that the AAP is an instrument for measuring attachment whose correlations with other methods for measuring attachment characteristics are still unknown. Additionally, we did not use all items of the scales “hyperactivity” and “externalizing problems” in our study because we wanted to minimize the burden on the mothers. Therefore, a general classification of symptom severity for these two scales was not possible and our results from the SDQ cannot be compared with the results of other studies. Furthermore, other variables might play a role in the calculated path way, that have not been included and therefore have confounded the results. For example, single motherhood or factors associated with the father could have an influence on parenting and the child`s mental health. The major limitation of the study are the survey dates. Lack of coping strategies, harmful parental behavior, and the child's mental health were collected at the same time point. Therefore, no conclusion can be drawn as to which variable may have influenced which, which implies that causality cannot be assumed. Future research should attempt to obtain a larger sample with higher representativeness. In addition, surveying individual forms of coping strategies such as seeking help in the social environment could lead to even more differentiated results.

### Implications and future research

Our study suggests that there is a complex interplay between attachment, coping, parental behavior, and child's mental health. To summarize this complexity, there is a sequence of the presence of different risk factors in mothers (i.e., attachment style, lack of coping strategies, harmful parental behavior) that cumulatively contribute to their children showing effects in the area of their own mental health.

Moreover, we found that harmful parental behaviors or parental behavior of lower quality in particular can lead to externalizing, problematic behaviors in children and that the lack of engaging parental coping can lead to internalizing behavior problems in children. Our findings suggest that pandemic disasters and subsequent containment efforts create a condition, which, especially in connection with an insecure attachment representation of the mother, can negatively influence the mental health of the children. In this context, parental coping strategies and parental behavior seem to be the most important starting points for appropriate interventions.

Because of the increased dependence of children on their parents for stress regulation and the influence of parental stress on children's mental health, special response strategies are needed to address the mental health needs of young children and their families. Pandemic mitigation measures must take these needs into account. Because pandemic disasters are unique and there are no held-forward interventions for prolonged support and recovery our findings reinforce existing calls (51, 52) to expand preventive services to promote and maintain stress coping skills for both children and parents in order to maintain children's mental health in times of crisis. For example, Rauchfuß (53) already examined the topic of resource-based intervention in pregnancy in the context of preventing stress and thus preterm birth. Such an intervention, adapted to the living environment of mothers, would be a conceivable step towards improving their coping strategies.

## Conclusion

In this study, we showed the role of intrafamilial resources (e.g., secure attachment, engaged coping) on children's mental health and that the pandemic appeared to have a particularly negative impact on mothers with an insecure attachment style and thus on their children. This also revealed that externalizing behavior problems in children are predicted primarily by harmful parental behavior, whereas internalizing behavior problems depend primarily on parental coping ability. Therefore, tailored interventions for families should be offered that both focus on the different types of mental health problems in children and support parents in their coping skills as well as in their parental skills.

## Data Availability

The original contributions presented in the study are included in the article/Supplementary Material, further inquiries can be directed to the corresponding author/s.
